# Heterologous mannitol-1-phosphate dehydrogenase gene over-expression in Parachlorella kessleri for enhanced microalgal biomass productivity

**DOI:** 10.1186/s43141-022-00322-7

**Published:** 2022-02-28

**Authors:** Jayant Pralhad Rathod, Chaitali Vira, Arvind M. Lali, Gunjan Prakash

**Affiliations:** 1 DBT-ICT Centre for Energy Biosciences, Institute of Chemical Technology, Mumbai, India; 2 ADT’s Shardabai Pawar Mahila Arts, Commerce & Science College, Baramati, Maharashtra India; 3 Department of Chemical Engineering, Institute of Chemical Technology, Mumbai, India

**Keywords:** Microalgae, Mannitol, Abiotic stress, High-light, ROS

## Abstract

**Background:**

Microalgae have tremendous potential in CO_2_ sequestration, bioenergy, biofuels, wastewater treatment, and high-value metabolites production. However, large-scale production of microalgae is hampered due to photo-inhibition in outdoor cultivation. Mannitol, as an osmolyte, is known to relieve the stress produced under different abiotic stress conditions during the growth of a photosynthetic organism.

**Results:**

In the present study, *Mannitol-1-phosphate 5-dehydrogenase* (*Mt1D*) was over-expressed to study the effect of mannitol over-production in *Parachlorella kessleri* under high-light induced stress. Over-expression of *Mt1D* led to 65% increased mannitol content in the transformed *P. kessleri* compared to that of wild type. Mannitol transformant demonstrated > 20-fold reduction in reactive oxygen species generation and 15% higher biomass productivity when grown in outdoor cultivation with high-light irradiance of 1200 μmol photons m^−2^ s^−1^.

**Conclusions:**

The current study establishes that a higher mannitol concentration provides stress shielding and leads to better acclimatization of transgenic microalgae against high-light generated stress. It also led to reduced ROS generation and improved growth of microalga under study. Thus, overexpression of the *Mt1D* gene in microalgae can be a suitable strategy to combat high-light stress.

## Background

Microalgae play an instrumental role in CO_2_ sequestration, thus helping mitigate greenhouse gases [1]. They are known to be the most promising organisms for “high volume-low cost” commodities like biofuel and “high cost-low volume” products like carotenoids, omega-3 fatty acids, vitamins [2, 3]. Cultivating marine algae is less resource-intensive than that of freshwater one because it excludes the use of arable land and freshwater [4]. Outdoor cultivation in sunlight is the preferred method of cultivation due to the lower cost of cultivation at a large scale. However, high-light irradiance causes photo-inhibition due to the over-absorption of light energy beyond the microalgae capacity to use it for carbon fixation [5]. The excess light generates reactive oxygen species (ROS), which is detrimental for microalgal growth [5–7]. These cumulative factors hinder achieving economically feasible biomass productivity in outdoor cultivation. Closed cultivation of microalgae, using photobioreactor employing artificial light of low intensity has thus gained impetus in the last two decades. However, the technology has not yet matured to the level of commercial implementation [8]. Various engineering and biological solutions have thus been adopted to reduce the photo-inhibition in open cultivation. Scavenging ROS through compatible solutes or antioxidant enzymes is one of the primary mechanisms to mitigate abiotic stress resistance in microalgae [9, 10]. Compatible solutes like trehalose, glyceinebetain, and mannitol also demonstrate ROS scavenging activity, provide osmoregulation, act as energy storage, and reduce power sink molecules. Mannitol, a sugar alcohol, is known to have all these properties and has been reported to be functional in plants against multiple abiotic stresses [11, 12] by over-expression of mannitol pathway genes.

Mannitol-1-phospahate converted from fructose-6-phosphate to mannitol-1- phosphate using mannitol-1-phosphate dehydrogenase (Mt1D) enzyme and subsequently convert it to mannitol by mannitol-1-phosphatase (M1pase) enzyme [13] by microorganisms. Overexpression of *Mt1D* gene in wheat, eggplant, sorghum, maize, and peanut has been shown to impart tolerance to salinity, drought stress and/or improved plant height/biomass [14]. The *M1pase* over-expression has also led to mannitol over-production in a cyanobacterium and *Escherichia coli* [11, 15]. The presence of mannitol synthesis genes has been reported in micro and macroalgae [16–18] like plants and other organisms. Therefore, enhancing mannitol content by overexpression of either one or both the genes might be helpful in the mitigation of light-induced stress in microalgae.

*P. kessleri* is a marine alga with high lipid content and thus is suitable for biodiesel (high volume–low cost) production at a large scale [19, 20]. It is also being cultivated to synthesize lutein (low volume–high cost) [21]. Therefore, it is an ideal species to be used in an algal bio-refinery for the overall sustainability of algal cultivation for biofuel. In outdoor cultivation, *P. kessleri* has been reported to undergo photo-inhibition due to high-light irradiance, resulting in retardation of cell growth [22] and lower biomass productivity in outdoor cultivation. In the present study, the first gene of the mannitol pathway (*Mt1D*) was overexpressed in *P. kessleri* to study its impact on the cell growth under high-light irradiance, i.e., photo inhibiting conditions.

## Methods

### Culture, media, and growth conditions

Walne’s medium was used to grow and maintain wild-type (WT) *P. kessleri*. For transformation, *P. kessleri* was shifted to tris acetate phosphate (TAP) medium [22], and transformant was maintained on solid TAP agar containing 15 mg l^−1^ hygromycin (Hi-media, India). Both WT and transformant cultures were maintained on Walne’s medium under a continuous light intensity of 75 μmol photons m^−2^ s^−1^ on an incubator shaker (Eppendorf) at 25 °C and 100 rpm.

### Plasmid construction and transformation

*Mt1D* gene (GenBank DQ660889.10) isolated from the genomic DNA of *E. coli* was a generous gift [15]. The full-length cloned gene was amplified by polymerase chain reaction (PCR) using forward primer 5′-CACCATGAAAGCATTACATTTTGG-3′ and reverse primer 5′-TTATTGCATTGCTTTATAAGCG-3′. PCR was executed at initial denaturation of 94 ^○^C for 5 min followed by 34 cycles of denaturation at 94 ^○^C for 1 min, annealing at 52 ^○^C for 1 min and extension at 72 ^○^C for 2 min with a final extension of 5 min at 72 ^○^C. The PCR products were analyzed on 1% agarose gel.

The extracted PCR product from the above reaction containing CACC at 5′ forward side was cloned into pENTR vector (Invitrogen) using the manufacturer’s protocol. Gateway® technology was used to clone *Mt1D* gene in pH7RWG2.0 vector with the help of Gateway® LR Clonase® II kit using manufacturer’s protocol (Invitrogen). pH7RWG2.0 vector harbouring *Mt1D* gene was transformed in *P. kessleri* using particle bombardment (Bio-Rad) protocol as described earlier [22]. In short, 1 mg gold carrier particle (Seashell Technology, USA) of approximately 550 nm were coated with 2 μg of plasmid and bombarded at a pressure of 900 psi and a distance of 6 cm. Post bombardment, the plates were kept in the dark for overnight incubation followed by scraping of cells and plating the cells onto selective TAP agar medium containing 15 mg l^−1^ hygromycin for 3–4 weeks.

### Molecular analysis

USING TAKARA KIT, genomic DNA was isolated from 1 × 10^7^cells of transformed and wild-type *P. kessleri* culture [22]. The presence of *Mt1D* gene in the genomic DNA of transformant *P. kessleri* was confirmed by PCR. The plasmid harbouring the *Mt1D* gene was used as a positive control and the PCR products were analyzed on 1% agarose gel. SDS PAGE was performed by extracting total soluble protein from WT and transformed *P. kessleri* cells. Total soluble protein extracts were prepared using 10 ml of WT and transformed *P. kessleri* cells. The cells were harvested by centrifugation at 10,000 rpm for 5 min and resuspended in 200 μl extraction buffer (50 mM HEPES, 5 mM MgCl_2_, 1 mM EDTA, 1 mM EGTA, 10% glycerol, 1% Triton-X 100, 2 mM benzamidine hydrochloride, 2 mM 6-aminocaproic acid, 0.5 mM PMSF, 10 mM DTT). The samples were denatured at 95 °C for 5 min. The extracted proteins were separated on 12% acrylamide gel by SDS-PAGE and the proteins were stained by standard silver staining protocol [23].

### High-light stress experiment

As mentioned earlier, *P. kessleri* WT and transformant were grown in a specially designed environmental chamber (EC) [7]. EC is made up of glass, and the temperature was maintained at 25 °C using the air conditioner. The cultures were exposed to diurnal variations of sunlight as the sole source of light, with natural light intensity reaching up to 1200 μmol photons m^−2^ s−^1^ during the period of experimentation. All the experiments were done in triplicates.

### Growth, nitrate, and pigment analysis

Growth of the algal cultures was monitored by measuring their optical density at 750 nm using a UV-Visible spectrophotometer (UV 2550, Shimadzu) at a regular interval of 24 h. At the end of the experiment, dry cell weight of the culture was determined by centrifuging the culture broth and washing the cell pellet thrice with distilled water to remove any traces of medium salts. The washed pellet was dried at 70 °C until a constant weight was obtained. For nitrate consumption analysis, cells were centrifuged at 8000 rpm and supernatant was analyzed by spectrophotometric method at 220 nm [24]. The standard graph was prepared using sodium nitrate (2–20 mg l^−1^) solution. Chlorophyll a, chlorophyll b, and total carotenoids were determined as described by Kumari et al. 2020 and Rathod et al. 2020 at stationary phase [25, 26]. In short, the algal cultures were centrifuged at 5000 rpm to get the pellet. The supernatant was removed, and 99.9% methanol was added to the pellet for pigments extraction. The samples were kept in dark for 30 min. The samples were centrifuged, and supernatant was taken to measure the absorbance at 665, 652, and 480 nm. Standard equations were used to calculate the chlorophyll a, chlorophyll b, and total carotenoids [25, 26].

### Quantification of mannitol production

Mannitol was extracted from dried biomass of *P. kessleri* cultures (both WT and transformant) at stationary phase using the method described by Jang et al. 2003 [27]. Briefly, dried biomass was crushed in liquid nitrogen and resuspended in 1 ml of distilled water. This sample was kept in boiling water bath for 15 min which was then cooled and filtered through 0.2 μm filter.

Mannitol content was determined by HPLC on Agilent 1200 series (Agilent Technologies, USA) using Bio-Rad Aminex HPX-87H Column (250 × 4.6 mm) with 5 mM sulphuric acid in the mobile phase at a flow rate of 0.6 mL/min. Column temperature was maintained at 45 °C. The eluted samples were detected by RI detector [15]. Commercially available mannitol (Sigma) was used as the standard.

### Estimation of reactive oxygen species and membrane damage

The extent of high-light stress in *P. kessleri* was determined towards the end of the growth phase. ROS presence was determined by colorimetric estimation of extracellular hydrogen peroxide (H_2_O_2_) production according to the method detailed by Rathod et al., 2016 [22]. The extent of membrane damage due to generation of the ROS was assessed by estimating the intracellular malondialdehyde (MDA) content. Thiobarbituric acid (TBA) assay was employed for MDA content determination as per the protocol described by Rathod et al. 2016 [22].

### Statistical analysis

Transformants and WT analysis were performed in triplicates. Collected data were compared using Student’s *t* test. Statistical analysis were performed using Microsoft excel.

## Results

### Overexpression of Mt1D

**Fig. 1:**
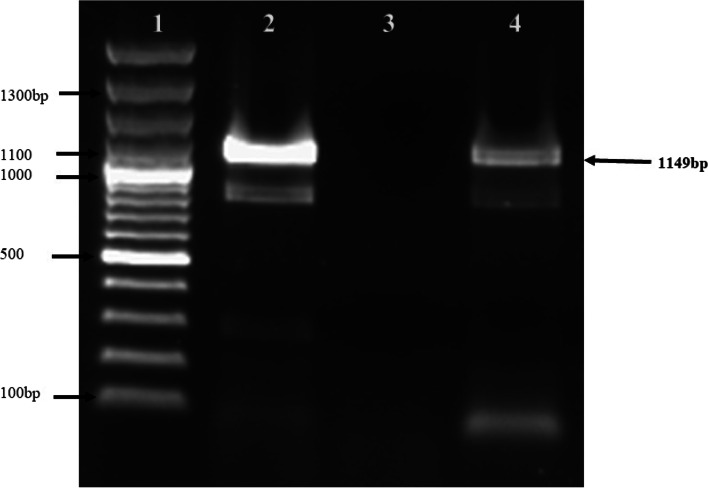
PCR analysis for *Mt1D* gene with gene-specific primers in wild-type and transformed *P. kessleri*. Lane 1: 100 bp ladder (from bottom to top 1000 bp, 1100 bp, 1200 bp, and 1300 bp), lane 2: PCR amplicon in positive control, lane 3: amplicon of wild-type *P. kessleri*, and lane 4: PCR amplicon of *Mt1D* transformant

The *Mt1D* gene (1149 bp) was cloned downstream of the cauliflower mosaic virus (CaMV) 35S promoter. Transformation of *Mt1D* gene was carried out in *P. kessleri* by particle bombardment method. In this case, only one transformant (M1) for *P. kessleri* was observed. The single *P. kessleri* mannitol expressing transformant (M1) was confirmed by PCR using *Mt1D* gene-specific primers as depicted in Fig. 1.

**Fig. 2:**
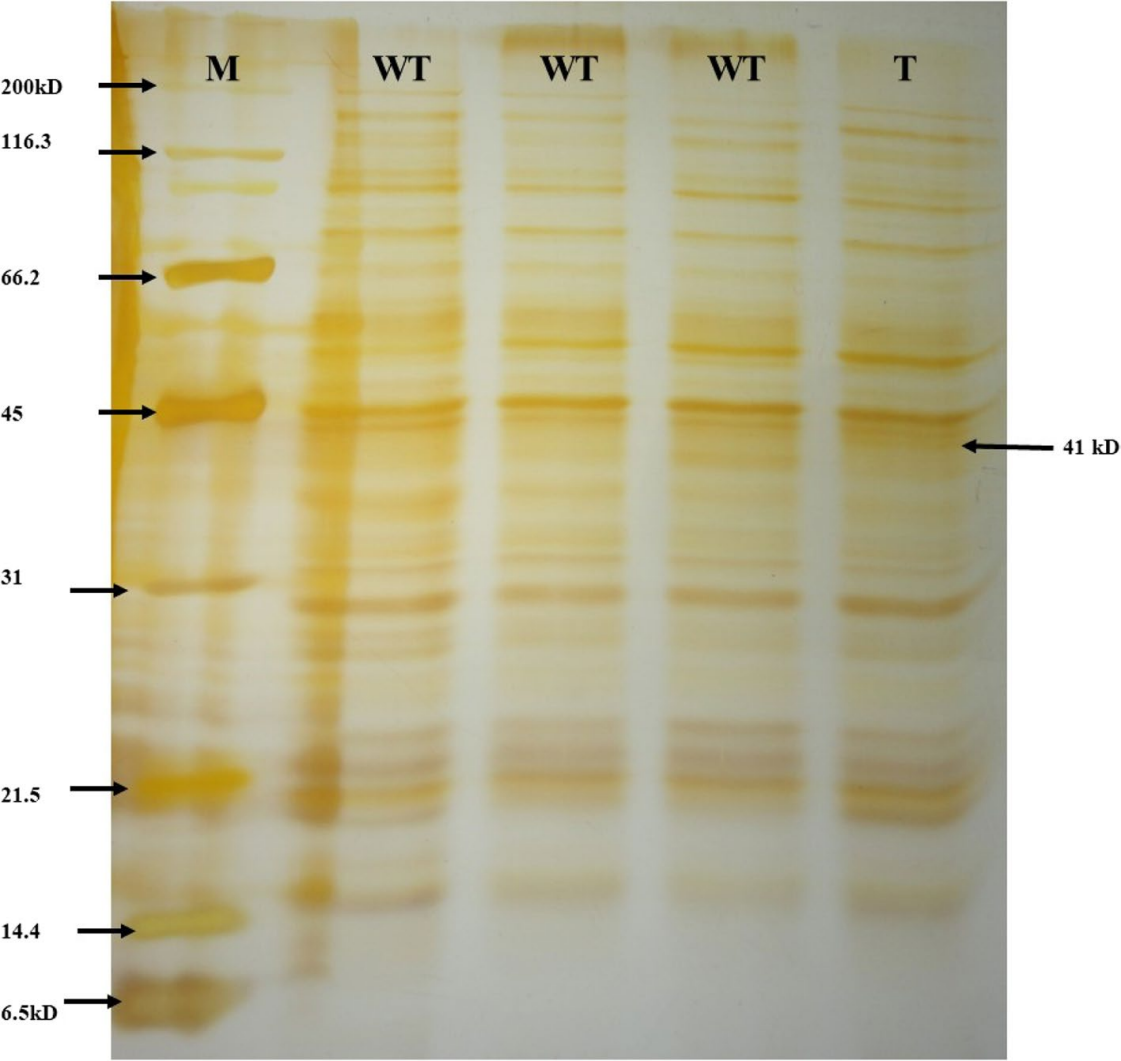
SDS PAGE analysis of wild-type and transformed *P. kessleri*. Lane 1: protein marker, Lanes 2, 3, and 4: total protein of WT and lane 5: total protein of transformant

M1 was sub-cultured under selection pressure till 20th generation to make sure its stability by PCR confirmation for *Mt1D* presence. The *Mt1D* gene codes for 382 amino acids, translating to 41.15 kDa of protein (GenBank: ABG54389.1). The presence of ~ 41 kDa band in transformed *P. kessleri* and its corresponding absence in wild-type culture (Fig. 2) indicated the successful integration of heterologous *Mt1D* gene and its successful translation to the corresponding protein in *P. kessleri*.

### Comparative study of wild-type and transformant under natural high light

**Fig. 3:**
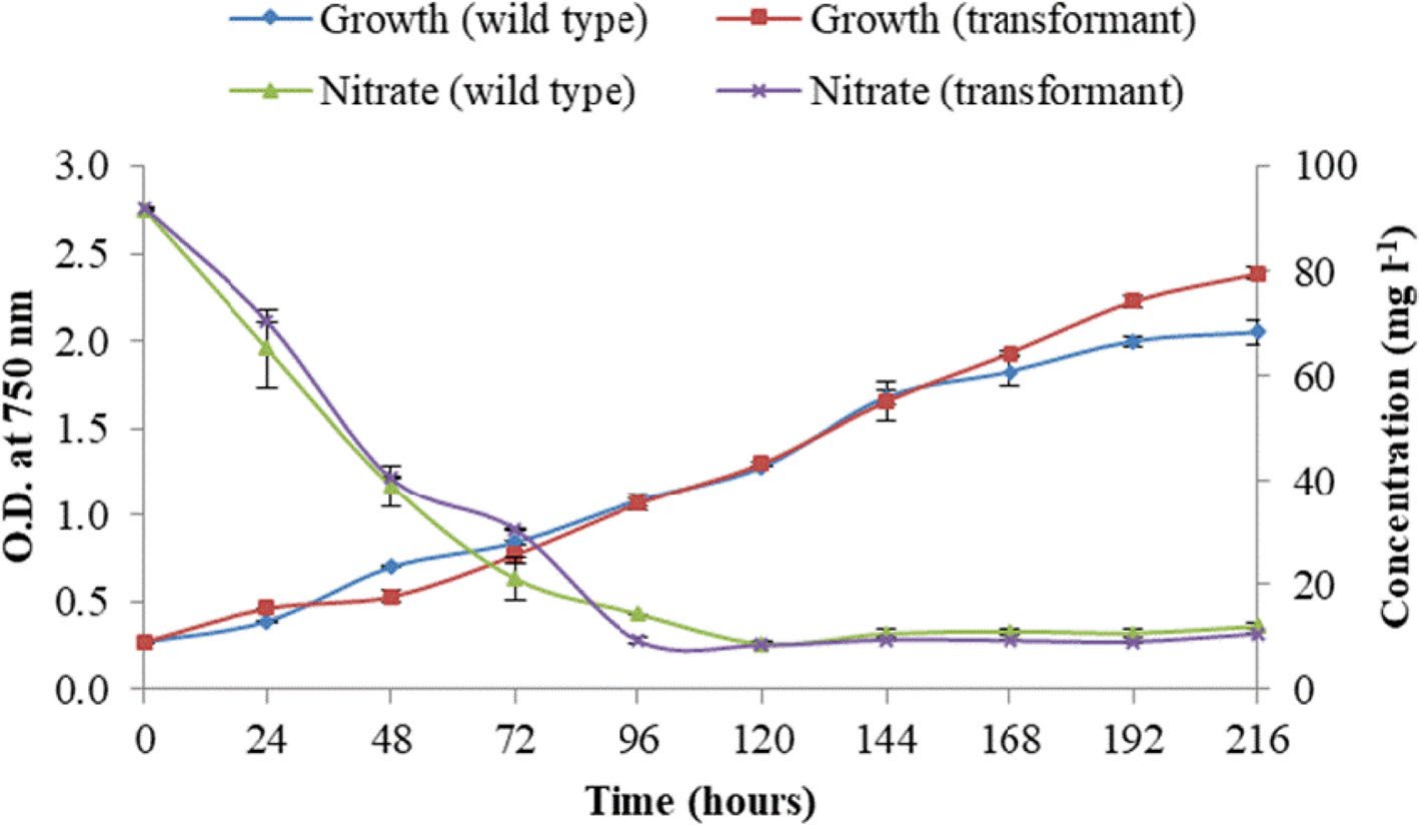
Growth study and nitrate uptake profile of transformed and wild-type *P. kessleri* under natural light of 0–1200 μmol photons m^−2^ s^−1^. Values are mean ± standard deviation (*n* = 3). Student’s *t* test was significant at *P* < 0.05 except for nitrate content which was significant at *P* < 0.09

**Fig. 4:**
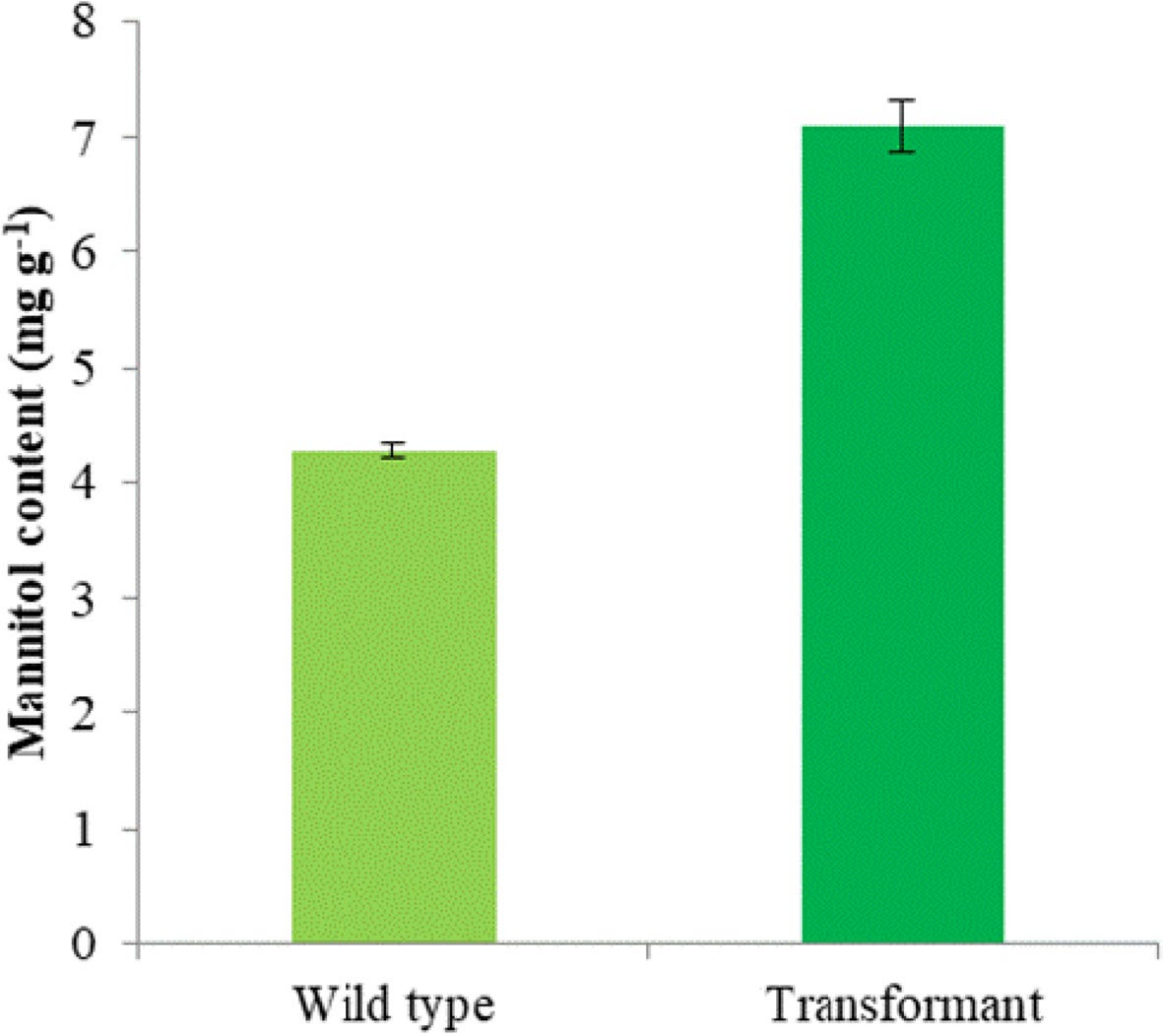
Mannitol content of wild-type and transformed *P. kessleri* in the light regime up to 1200 μmol photons m^−2^ s^−1^. Values are mean ± standard deviation (*n* = 3). Student’s *t* test was significant at *P* < 0.05

WT and M1 were subjected to light stress by cultivating them under natural light irradiance of 1200 μmol photons m^−2^ s^−1^ in EC. Under high-light irradiance, the optical density of wild type and M1 was similar until log phase (144 h) as depicted in Fig. 3. However, WT growth was super succeeded by M1 when the cultures started entering in the stationary phase. *Mt1D*-expressing *P. kessleri* culture displayed a 65% increase in mannitol content (Fig. 4) compared to that of WT. Concurrently, M1 exhibited 13% increased nitrate consumption which also correspondent to the similar percentage increase in biomass productivity (Fig. 3). The absolute amount of mannitol formed in *P. kessleri* WT, as well as the transformant (Fig. 4), was found to be higher. A drastic reduction (~ 20 fold) in H_2_O_2_ content release was obtained in M1 compared to WT when both were grown at 1200 μE (Table 1). A > 1.5-fold decrease in the internal concentration of MDA was obtained for M1 as compared to that of WT cells (Table 1).

**Table 1:** Comparison of stress parameters for wild-type and transformed *P. kessleri* in the light regime of 0–1200 μmol photons m^−2^ s^−1^

Stress parameters	Wild type	Transformant
**ROS (nM/10^4 cells)**	6.77 ± 0.22	0.33 ± 0.07
**MDA (μM g**^**−1**^**)**	278.18 ± 27.6	163.82 ± 3.99

Values are mean ± standard deviation (*n* = 3)

Student’s *t* test was significant at *P* < 0.05

An overall increase in pigment content was also observed for M1 compared to that of WT *P. kessleri* for both chlorophyll a (31%) and b (16%) as well as for the total carotenoids (8%) concentration as depicted in Table 2. The reduced chlorophyll antenna size of M1 compared to that of WT (indicated by higher chlorophyll a/b ratio) was observed.

**Table 2:** Pigments studies of wild-type and transformed *P. kessleri* in the light regime up to 1200 μmol photons m^−2^ s^−1^

Pigments	Wild type	Transformant
**Carotenoids (mg g**^**−1**^**)**	2.51 ± 0.27	2.71 ± 0.07
**Chlorophyll a (mg g**^**−1**^**)**	4.62 ± 0.43	6.04 ± 0.30
**Chlorophyll b (mg g**^**−1**^**)**	1.57 ± 0.32	1.82 ± 0.15
**Chlorophyll a/b**	2.97 ± 0.33	3.32 ± 0.12

Values are mean ± standard deviation (*n* = 3)

## Discussion

### Overexpression of Mt1D

CaMV 35S promoter is a commonly used promoter and has been used to express varied genes in different microalgae like *Dunaliella bardawil*, *D salina, Chlorella ellipsoidea*, *C. vulgaris*, and *Haematococcus pluvialis* [28–30]. CaMV 35S promoter has also been found to be functional in *P. kessleri* [31].

Only one transformant was observed in *Mt1D* transformation. This contrasted with the earlier report where higher transformation efficiency was reported for Trehalose phosphate phosphatase gene expression in *P. kessleri* using the same methodology [31]. In the present case, the low transformation efficiency of *Mt1D* could be attributed to the source of the gene (*E. coli*) which was prokaryotic in origin. The eukaryotic origin genes have led to more transformants in microalgae, including *P. kessleri* [22, 32, 33]. The codon optimization of *Mt1D* would have helped increase the transformation efficiency however could not be attempted because of the absence of codon optimization table for *P. kessleri*.

The *Mt1D* was successfully transformed into *P. kessleri* and was confirmed by PCR using *Mt1D* gene-specific primers and was found to be stable until the 20th generation. Transformation was further confirmed at protein expression level by SDS PAGE analysis.

### Comparative study of WT and transformant under natural high light

Mannitol is a well-known osmolyte that protects the cell against salt, drought, and chilling stress in different organisms [14, 18, 34, 35]. Over-expression of *Mt1D* in the *Solanum tuberosum* resulted in fresh weight and height enhancement [36]. The amount of mannitol formed in *P. kessleri* WT and transformant was much higher than that of transgenic petunia, tomato, and wheat plants over-expressing *Mt1D* gene [28, 37, 38]. This higher amount could be due to the marine origin of *P. kessleri*.

ROS generation and cellular lipid membrane damage were measured to investigate oxidative stress management by over-expression of mannitol in transformed *P. kessleri* cultures under high light stress conditions. Environmental stresses such as drought, salinity, and low temperature create ROS, which is mitigated by activating various oxidases and peroxidases in microalgae [39]. In marine microalgae, H_2_O_2_ has been reported to cause inhibition of photosynthesis enzymes [40]. The protective role of mannitol in dealing with hydroxyl radicals has also been reported in Petunia. Mannitol was found to react with hydroxyl radicals to form a mannitol radical which was then converted to mannose in the presence of oxygen, thus protecting the cell from oxidative damage [37].

The excessive generation of intracellular ROS due to oxidative stress also causes lipid peroxidation of cell membrane resulting in MDA synthesis [41]. Reduction in both H_2_O_2_ and MDA content indicated that over-expression of *Mt1D* gene which resulted in increased mannitol production facilitated stress relief in transformed *P. kessleri* cells. A higher reduction in H_2_O_2_ compared to that of MDA was significant because its half-life is more than other ROS molecules, and it diffuses faster through the membranes, causing severe damage leading to inhibition of multiple photosynthesis enzymes in marine microalgae [40]. Hema et al. 2014 have also observed increased resistance to oxidative stress linked to a reduction in superoxide radical production in transgenic plants by over-expression of *Mt1D* gene [12].

In transgenic tomato, over-expression of bacterial *Mt1D* gene increased total chlorophyll content against multiple abiotic stresses [28]. One of the possible reasons for the increase in chlorophyll content in M1 could be the requirement of NADH, the reducing power for mannitol-1-phosphate dehydrogenase enzyme to synthesize mannitol-1-phosphate from fructose-6-phosphate [11]. The requirement of NADH might have been fulfilled by increasing the overall pigment composition to enhance photosynthesis by chlorophyll pigments in case of *P. kessleri* and transgenic tomato [28].

The reduced chlorophyll antenna size of M1 was observed, suggested it to be a better performer under high-light intensities due to decreased absorption of incident light. Reduced chlorophyll antenna size phenomenon was also obtained in *P. kessleri* when *Trehalose Phosphate Synthase* was over-expressed [22]. Reduced chlorophyll antenna size is preferred under high-light growth conditions and is helpful to achieve higher growth densities in microalgae [7, 42].

In summary, mannitol over-production by over-expression of the first gene of mannitol pathway led to increasing mannitol production and pigments which could combat high-light irradiance generated stress. The cumulative effects of all the effects resulted in increased biomass productivity of *P. kessleri* by 15% under high-light stress conditions in *Mt1D* transformant.

The mechanism of mannitol action against stress release is not fully understood. However, few hypotheses relating to membrane lipid and protein stabilization, scavenging of free oxygen molecules, and maintaining the turgor of cells at low water activity have been proposed in plants [43]. There are very few reports of osmolytes over-expression in microalgae and cyanobacteria. Choline oxidase, glycinebetaine, and trehalose have been reported to enhance growth under different abiotic stress conditions in the microalgae [31, 44, 45]. To the best of our knowledge, before the present work, the effect of over-production of mannitol against light induced abiotic stress has not reported in microalgae.

## Conclusion

Over-expression of *MtlD* gene in marine and oleaginous *P. kessleri* resulted in higher levels of mannitol accumulation and protection under high-light induced stressed growth conditions. Thus, mannitol over-expression can be an efficient way to mitigate light-mediated abiotic stress in microalgae.

## Data Availability

Not applicable

## Abbreviations

CaMV: Cauliflower mosaic virus
Mt1D: Mannitol-1-phosphate dehydrogenase
M1pase: Mannitol-1-phosphatase
ROS: Reactive oxygen species
MDA: Malondialdehyde
TBA: Thiobarbituric acid
